# Antagonists of growth hormone-releasing hormone (GH-RH) inhibit IGF-II production and growth of HT-29 human colon cancers

**DOI:** 10.1054/bjoc.2000.1223

**Published:** 2000-04-27

**Authors:** K Szepeshazi, A V Schally, K Groot, P Armatis, G Halmos, F Hebert, B Szende, J L Varga, M Zarandi

**Affiliations:** Endocrine, Polypeptide and Cancer Institute, Veterans Affairs Medical Center, New Orleans, Louisiana, USA; Department of Medicine, Tulane University School of Medicine, New Orleans, Louisiana, USA; 1st Institute of Pathology and Experimental Cancer Research, Semmelweis University School of Medicine, Budapest, Hungary

**Keywords:** GH-RH antagonists, colon cancer, IGF-I, IGF-II, IGF-I receptor, AgNOR, apoptosis

## Abstract

Insulin-like growth factors (IGFs) I and II are implicated in progression of various tumours including colorectal carcinomas. To interfere with the production of IGFs, we treated male nude mice bearing xenografts of HT-29 human colon cancer with various potent growth hormone-releasing hormone (GH-RH) antagonists. Twice daily injections of antagonist MZ-4-71, 10 μg intraperitoneally or 5 μg subcutaneously (s.c.) resulted in a significant 43–45% inhibition of tumour growth. Longer acting GH-RH antagonists, MZ-5-156 and JV-1-36 given once daily at doses of 20 μg s.c. produced a 43–58% decrease in volume and weight of cancers. Histological analyses of HT-29 cancers demonstrated that both a decreased cell proliferation and an increased apoptosis contributed to tumour inhibition. GH-RH antagonists did not change serum IGF-I or IGF-II levels, but significantly decreased IGF-II concentration and reduced mRNA expression for IGF-II in tumours. In vitro studies showed that HT-29 cells produced and secreted IGF-II into the medium, and addition of MZ-5-156 dose-dependently decreased IGF-II production by about 40% as well as proliferation of HT-29 cells. Our studies demonstrate that GH-RH antagonists inhibit growth of HT-29 human colon cancers in vivo and in vitro. The effect of GH-RH antagonists may be mediated through a reduced production and secretion of IGF-II by cancer cells. © 2000 Cancer Research Campaign


British Journal of Cancer (2000) 82(10), 1724–1731
© 2000 Cancer Research Campaign
DOI: 10.1054/ bjoc.2000.1223, available online at http://www.idealibrary.com on IDEAL®

### Antagonists of growth hormone-releasing hormone (GH-RH) inhibit IGF-II production and growth of HT-29 human colon cancers

K Szepeshazi[1,2], AV Schally[1,2], K Groot[1], P Armatis[1], G Halmos[1,2], F Hebert[1], B Szende[3], JL Varga[1,2] and M Zarandi[1,2]

[1]Endocrine, Polypeptide and Cancer Institute, Veterans Affairs Medical Center, New Orleans, Louisiana, USA; [2]Department of Medicine, Tulane University School of Medicine, New Orleans, Louisiana, USA; [3]1st Institute of Pathology and Experimental Cancer Research, Semmelweis University School of Medicine, Budapest, Hungary

**Summary** Insulin-like growth factors (IGFs) I and II are implicated in progression of various tumours including colorectal carcinomas. To interfere with the production of IGFs, we treated male nude mice bearing xenografts of HT-29 human colon cancer with various potent growth hormone-releasing hormone (GH-RH) antagonists. Twice daily injections of antagonist MZ-4-71, 10 μg intraperitoneally or 5 μg subcutaneously (s.c.) resulted in a significant 43–45% inhibition of tumour growth. Longer acting GH-RH antagonists, MZ-5-156 and JV-1-36 given once daily at doses of 20 μg s.c. produced a 43–58% decrease in volume and weight of cancers. Histological analyses of HT-29 cancers demonstrated that both a decreased cell proliferation and an increased apoptosis contributed to tumour inhibition. GH-RH antagonists did not change serum IGF-I or IGF-II levels, but significantly decreased IGF-II concentration and reduced mRNA expression for IGF-II in tumours. In vitro studies showed that HT-29 cells produced and secreted IGF-II into the medium, and addition of MZ-5-156 dose-dependently decreased IGF-II production by about 40% as well as proliferation of HT-29 cells. Our studies demonstrate that GH-RH antagonists inhibit growth of HT-29 human colon cancers in vivo and in vitro. The effect of GH-RH antagonists may be mediated through a reduced production and secretion of IGF-II by cancer cells. © 2000 Cancer Research Campaign

**Keywords**: GH-RH antagonists; colon cancer; IGF-I; IGF-II; IGF-I receptor; AgNOR; apoptosis

Colorectal cancer is the second most frequent cause of death from malignancies in the Western world (Hardcastle, 1997). The survival of colon cancer patients could be substantially improved by early diagnosis, but many colorectal cancers are detected at a late stage when surgery cannot cure the disease. At least 40% of patients with colorectal cancer develop metastases during the course of their illness (Labianca et al, 1997). Chemotherapy alone or combined with radiotherapy can be used as a treatment approach for advanced disease or as an adjuvant therapy to surgery (Labianca et al, 1997). However, none of these approaches is highly effective against disseminated colonic cancer (Magnusson et al, 1995), and the search for new treatment modalities must continue.

In the past few years, more findings have been accumulated supporting the role of growth hormone (GH)–insulin-like growth factor (IGF) I axis in growth of colorectal cancers (Singh and Rubin, 1993; Pollak et al, 1987). Increased colon epithelial cell proliferation and a higher risk of developing colonic and other cancers were demonstrated in patients with acromegaly (Orme et al, 1998), showing that elevated serum GH and IGF-I levels may be associated with a higher incidence of colon cancers and various other malignancies (Pollak et al, 1987, 1989; Pollak and Schally, 1998; Ma et al, 1999). In addition to IGF-I, IGF-II may also play a major role in progression of colon tumours (Tricoli et al, 1986; Lahm et al., 1992; Macaulay, 1992). IGF-II mRNA was found to be overexpressed in about one-third of colon cancers while it was not detectable in normal colonic mucosa cells (Michell et al, 1997). IGF-II may have a regulatory role in the proliferation and differentiation of human colonic carcinoma cells (Garrouste et al, 1997). Colon cancer cells also produce specific proteases that degrade IGF binding proteins (IGFBP) secreted by these cells, thus decreasing the protective effect of IGFBPs against activation of the IGF-I receptor (IGF-IR) (Michell et al, 1997).

In view of the involvement of IGF-I and II in growth of various tumours, we synthesized a series of potent antagonists of growth hormone-releasing hormone (GH-RH) which interfere with the GH-RH-GH axis, reduce the secretion of IGF-I by the liver and decrease the level of IGF-I or -II in tumours (Schally, 1994; Zarandi et al, 1994, 1997; Schally et al, 1998; Varga et al, 1999). These analogues inhibit growth of various experimental tumours (Pinski et al, 1995; Lamharzi et al, 1998; Schally et al, 1998; Kiaris and Schally, 1999). In the present study, we evaluated the effects of five GH-RH antagonists on growth of HT-29 human colon cancers in vivo and in vitro.

Received 26 July 1999
Revised 3 January 2000
Accepted 9 January 2000

Correspondence to: AV Schally, Endocrine, Polypeptide and Cancer Institute, VA Medical Center, 1601 Perdido St, New Orleans, LA 70112–1262 USA

### MATERIALS AND METHODS

### Peptides

GH-RH antagonists [Ibu-Tyr$^1$,D-Arg$^2$,Phe(4-Cl)$^6$,Abu$^{15}$,Nle$^{27}$]hGH-RH(1-28)Agm (MZ-4-71),[PhAc-Tyr$^1$,D-Arg$^2$,Phe(4-Cl)$^6$,Ala$^{15}$,Nle$^{27}$]hGH-RH(1-29)$NH_2$ (MZ-5-186),[PhAc-Tyr$^1$,D-Arg$^2$,Phe(4-Cl)$^6$,Abu$^{15}$,Nle$^{27}$]hGH-RH(1-28)Agm(MZ-5-156),[PhAc-Tyr$^1$,

D-$Arg^{2}$,Phe(4-Cl)$^{6}$,$Arg^{9}$,$Abu^{15}$,$Nle^{27}$,D-$Arg^{29}$]hGH-RH(1-29)$NH_2$(JV-1-10), and [PhAc-$Tyr^{1}$,D-$Arg^{2}$,Phe(4-Cl)$^{6}$,$Arg^{9}$,$Abu^{15}$,$Nle^{27}$,D-$Arg^{28}$,$Har^{29}$]hGH-RH(1-29)$NH_2$ (JV-1-36) were synthesized in our laboratory by solid phase methods (Zarandi et al, 1994, 1997; Varga et al, 1999). The peptides were dissolved in 20 µl dimethylsulphoxide (DMSO) (Sigma, St Louis, MO, USA) and diluted with 10% propylene glycol in water.

### Animals and tumours

Male athymic nude mice (Ncr *nu/nu*), approximately 6 weeks old on arrival, were obtained from the National Cancer Institute (Frederick, MD, USA), and maintained under pathogen-limited conditions. All experiments were approved by the institutional ACUC and the procedures were essentially in accordance with UKCCCR guidelines for the welfare of animals in experimental neoplasia. The methods for culturing of HT-29 cells are described under in vitro studies. Xenografts were initiated by subcutaneous (s.c.) injection of $10^{7}$ cells into the right flank area of nude mice. Two-$mm^{3}$ pieces of the developed tumours were further transplanted s.c. into the experimental animals.

### Experimental protocol

Three in vivo experiments were performed. In Experiment I, the groups were treated as follows: (1) Control, injection vehicle only; (2) MZ-4-71, 10 µg twice daily; (3) MZ-5-186, 10 µg twice daily. Groups 2 and 3 consisted of nine mice, and Group 1 had 15 animals. The treatment was started 15 days after transplantation of tumours and the mice were injected intraperitonealy (i.p.) twice daily for 33 days. In Experiment II, the treatment started 7 days after transplantation of tumours: Group 1 control received vehicle only; Group 2 MZ-4-71 5 µg twice daily s.c. for 25 days. In Experiment III, five groups were formed as follows: (1) Control, vehicle only; (2) MZ-4-71; (3) MZ-5-156;(4) JV-1-10; (5) JV-1-36. The treatment was initiated 19 days after transplantation. The mice were injected once daily s.c. with 20 µg of analogues for 42 days. In Experiments II and III the groups consisted of ten animals. Tumour volume was measured weekly. At the end of the experiments, the mice were sacrificed under Metofane (Malinkrodt Vet., Mundelein, IL, USA) anaesthesia by exsanguination. In Experiment II, two mice in each group received 3 mg of bromodeoxyuridine (BrdU) (Sigma, St Louis, MO, USA) i.p. 2 h before sacrificing.

### Histological procedures

Samples were fixed in 10% buffered formalin. Specimens were embedded in Paraplast (Oxford Labware, St Louis, MO, USA). Mitotic and apoptotic cells were counted in sections stained with haematoxylin and eosin and their numbers per 1000 cells were accepted as the mitotic and apoptotic indices respectively. For demonstration of the nucleolar organizer region (NOR) in tumour cell nuclei, the AgNOR method was used as described (Szepeshazi et al, 1991). The number of AgNOR granules is an indicator of cell proliferation. The silver-stained black dots in 50 cells of each tumour were counted, and the AgNOR number per cell was calculated.

BrdU incorporated into cells advancing through S phase of cell cycle was detected in paraffin-embedded tumour tissue by immunohistochemistry. Tissue sections on silanated glass slides were treated with 1% hydrogen peroxide for 15 min and then with 1.0 N hydrochloric acid (HCl) at 60°C for 20 min. Incubation in the monoclonal anti-BrdU (Sigma) for 60 min at room temperature (RT) was followed by another incubation in biotin-conjugated anti-mouse IgG (Vector, Burlingame, CA, USA), 6 µg $ml^{-1}$, at RT for 45 min. ABComplex/HRP reagent (Dako, Carpinteria, CA, USA) was used for 30 min at RT, and the reaction was visualized with DAB (Sigma). The BrdU containing nuclei in ten high power microscopic fields were counted and their number per 1000 cells was accepted as BrdU index.

### Electron microscopy

For electron microscopy in Experiment II, 1 $mm^{3}$ pieces of tumour tissue were fixed in 2.5% glutaraldehyde for 1 h, postfixed in 2% $OsO_4$ for 1 h, and embedded into Polybed (Polysciences, Warrington, PA, USA). The ultrathin sections were counterstained with uranyl acetate and lead citrate, and studied using a JEM 100 B electron microscope (JEOL, Tokyo, Japan).

### IGF-I receptor assay

Receptor binding was characterized using sensitive in vitro ligand competition assay based on binding of [$^{125}$I]IGF-I (Amersham Pharmacia Biotech, Piscataway, NJ, USA) as radioligand to tumour membrane fractions (Pinski et al, 1995). In brief, membrane homogenates containing 50–120 µg protein were incubated in triplicate with 50–60 000 cpm radioligand and increasing concentrations ($10^{-12}$–$10^{-6}$ mol $l^{-1}$) of non-radioactive peptides as competitors in a total volume of 150 µl of binding buffer. At the end of the incubations, the reactions were terminated by adding ice-cold assay buffer to the tubes, and the bound ligand was separated from free ligand by centrifugation. Radioactivity in the pellet of each tube was counted in a gamma counter (Micromedic Systems, Huntsville, AL, USA). Non-specific binding was 1.4–3.3% of the incubation concentration of [$^{125}$I]IGF-I in all tumour samples examined. Protein concentrations were determined using a Bio-Rad protein assay kit (Bio-Rad Laboratories, Hercules, CA, USA). The LIGAND PC computerized curve-fitting program of Munson and Rodbard (1980) was used to evaluate types of receptor binding, the maximal binding capacity ($B_{max}$) of the receptor and the dissociation constant ($K_d$) values.

### Radioimmunoassays for GH, IGF-I and IGF-II

Serum GH was determined using materials provided by Dr AF Parlow (National Hormone & Pituitary Program, Torrance, CA, USA): mouse GH 10783B for standard iodination and anti-rat GH-RIA-5/AFP-411S. The methods used for determination of IGF-I and IGF-II levels in serum, colon tumour samples and cell culture media after acid–ethanol cryoprecipitation were described (Lamharzi et al, 1998). Briefly, 100 mg of tumour tissue was homogenized in 1 ml of homogenization buffer (0.05 M Tris–HCl/0.05 M magnesium chloride ($MgCl_2$)/30 µg $ml^{-1}$ bacitracin, pH 7.6) and centrifuged at 2000 ***g*** (20 min at 4°C). Protein determination in the supernatant was performed by using Bio-Rad (Hercules, CA, USA) protein assay kit. All serum and tumour homogenate samples were extracted by a modified acid-ethanol cryoprecipitation method. The extracted IGF-I in serum and tumour samples were measured by RIA using IGF-I (88-G4 Genentech, San Francisco, CA, USA) standard and antibody UB2-495 (a gift from LE Underwood and J Van Wyk, University of

*© 2000 Cancer Research Campaign*

**Table 1** Effect of treatment with various GH-RH antagonists on tumour volume and weight, IGF-II concentrations and IGF-II mRNA expression in HT-29 human colon cancers growing in nude mice

| Groups | Tumour volume ($mm^3$) | | Tumour weights (g) | IGF-II in tumours | |
|---|---|---|---|---|---|
| | Initial | Final | | Concentrations (pg 100 µg$^{-1}$ protein) | mRNA expression (ratio to β-actin) |
| Experiment I (dose 10 µg twice daily i.p.) | | | | | |
| 1. Control | 116 ± 31 | 5386 ± 1120 | 5.68 ± 1.25 | | |
| 2. MZ-4-71 | 127 ± 57 | 2985 ± 726[a] | 3.63 ± 0.97 | | |
| 3. MZ-5-186 | 122 ± 71 | 3657 ± 808 | 4.91 ± 0.94 | | |
| Experiment II (Dose 5 µg twice daily s.c.) | | | | | |
| 1. Control | 22 ± 7 | 9514 ± 1809 | 10.27 ± 2.04 | 408 ± 13 | |
| 2. MZ-4-71 | 23 ± 4 | 5402 ± 1331[a] | 7.53 ± 1.81 | 264 ± 10[a] | |
| Experiment III (dose 20 µg day$^{-1}$ s.c.) | | | | | |
| 1. Control | 70 ± 31 | 2117 ± 751 | 2.36 ± 0.84 | 353 ± 8 | 0.55 ± 0.09 |
| 2. MZ-4-71 | 64 ± 18 | 1953 ± 400 | 2.19 ± 0.46 | 280 ± 10 | 0.50 ± 0.13 |
| 3. MZ-5-156 | 57 ± 18 | 908 ± 195[a] | 1.01 ± 0.17[a] | 228 ± 24[a] | 0.31 ± 0.05[a] |
| 4. JV-1-10 | 67 ± 29 | 1194 ± 506[a] | 1.38 ± 0.58[a] | 297 ± 16 | NA |
| 5. JV-1-36 | 45 ± 12 | 890 ± 322[a] | 1.35 ± 0.48[a] | 238 ± 35[a] | 0.20 ± 0.03[a] |

Values are means ± s.e.m, [a]$P < 0.05$ vs control. NA = no data because of a technical problem. Experiments I and II were done in 1995.

North Carolina, Chapel Hill, NC, USA) was used at the final dilution of 1:14 000. IGF-II (Bachem, Torrance, CA, USA) standard and Amano mAB generated against rat IGF-II 10 µg ml$^{-1}$ were used at the final dilution of 1:14 285 (Amano International Enzyme, Troy, VA, USA).

### In vitro studies

The human colon cancer cell line HT-29 was purchased from ATCC (Rockville, MD, USA). The cells were routinely grown as a monolayer in McCoy 5A medium (Gibco, Grand Island, NY, USA) containing 5% fetal bovine serum (FBS) and antibiotics and antimycotics. HT-29 cells were seeded into 96-well microplates and cultured for 24 h. IGF-I (10 and 25 ng ml$^{-1}$), IGF-II (500 and 800 ng ml$^{-1}$), GH (5 and 25 ng ml$^{-1}$) and MZ-5-156 ($3 \times 10^{-7}$ and $3 \times 10^{-6}$ M) were added to the medium (t0). Controls received medium only. After 66 or 90 h, in vitro cell growth was estimated using the crystal violet assay (Bernhardt et al, 1992). The OD at 600 nm of each well was measured using a Beckman (Palo Alto, CA, USA) plate reader. Then, %T/C was calculated, where T = optical density ($OD_{600\ nm}$) of treated cultures and C = $OD_{600\ nm}$ of control cultures × 100 (t0 = $OD_{600}$ at start of experiment).

HT-29 cells were also cultured in serum-free medium containing McCoy 5A, 0.5% bovine serum albumin (BSA), 1 mM pyruvate, 50 µg ml$^{-1}$ transferrin and 1 mM $FeSO_4$. The IGF-I and IGF-II concentration of the medium was measured by radioimmunoassay (RIA), and the effect of $3 \times 10^{-7}$ and $3 \times 10^{-6}$ M of MZ-5-156, 25 ng ml$^{-1}$ of GH, 25 ng ml$^{-1}$ of IGF-I, 800 ng ml$^{-1}$ of IGF-II, on cell proliferation was studied using the crystal violet assay.

For measurement of IGF-I and IGF-II in media of HT-29 cells, samples were taken before seeding of cells (base medium) and 24 h after seeding, immediately before treatments (t0). The peptides were added to the media at concentrations described above (8 wells for each), the controls receiving medium only. Samples were taken 66 h and 138 h after addition of the peptides (t1 and t2 respectively). The experiment was done in duplicate.

### IGF-I and IGF-II mRNA expression in HT-29 cells and HT-29 tumours

HT-29 cells were harvested as described above 4 h after addition of MZ-5-156 to the medium. Total RNA was extracted from control and treated cells and tumour tissue samples as described (Lamharzi et al, 1998).

The methods of reverse transcription (RT) and polymerase chain reaction (PCR) amplification were also reported earlier (Lamharzi et al, 1998). Three micrograms of total RNA was reverse-transcribed using the RT-PCR Kit (Stratagene, La Jolla, CA, USA) according to manufacturer's instructions. PCRs for IGF-I, IGF-II and β-actin (internal control) were carried out in a final volume of 100 µl containing 10 mM Tris (pH 8.8), 50 mM potassium chloride, 1.5 mM $MgCl_2$, 0.2 mM of each dNTP, sense and antisense primers at a concentration of 0.12 µM each, and 2.5 U *Taq* 2000 polymerase (Stratagene). The primers for human IGF-I were (Lamharzi et al, 1998): 5′-ACATCTCCCATCTCTCTGGATTTC-CTTTTGC-3′ (sense) and 5′-CCCTCTACTTGCGTTCTTCA-AATGTACTTCC-3′ (antisense). The primers for human IGF-II were: 5′-AGTCGATGCTGGTGCTTCTCACCTTCTTGGC-3′ (sense) and 5′-TGCGGCAGTTTTGCTCACTTCCGATTGC-TGG-3′ (antisense). The primers for human β-actin were: 5′-TCATGAAGTGTGACGTGGAC-3′ (sense) and 5′-ACCGACTG-CTGTCACCTTCA-3′ (antisense). PCR was performed in a GeneAmp PCR System 2400 (Perkin-Elmer, Norwalk, CT, USA) using a step programme of 35 cycles of 95°C for 15 s and 60°C for 30 s for IGF-I and IGF-II. For β-actin, a step programme of 32 cycles of 94°C for 45 s, 60°C for 45 s and 72°C for 45 s was used. The reaction was finished with a final step of 72°C for 10 min. Negative controls containing RNA were used to confirm that the samples were not contaminated with genomic DNA. Ten microlitres of the PCR products were separated on 1.8% agarose gel and visualized by ethidium bromide on a UV transilluminator. The banded areas were scanned and quantified using an imaging densitometer (Bio-Rad).

 © 2000 Cancer Research Campaign

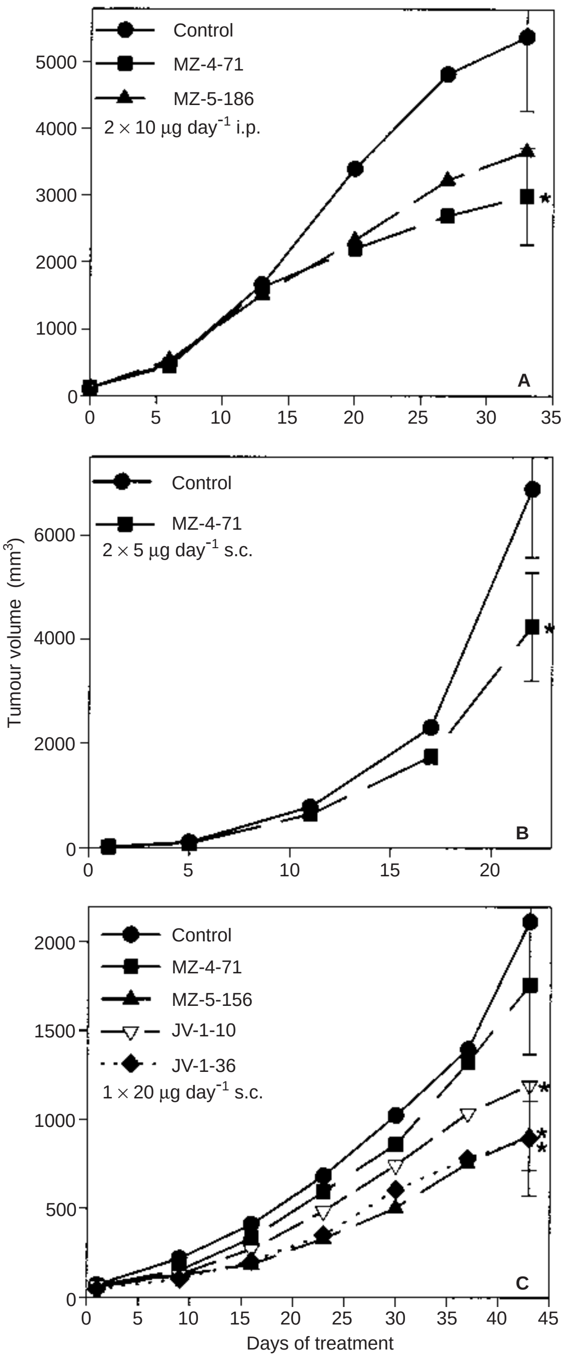

**Figure 1** Tumour volume changes of HT-29 human colon cancer xenografts in nude mice in Experiments I **(A)**, II **(B)** and III **(C)**. The mice were treated with daily injections of GH-RH antagonists i.p. (Experiment I) or s.c. (Experiment II and III). *$P$ < 0.05. The vertical bars show s.e.m. of selected groups

### Statistical methods

Statistical analyses of the data were performed using Duncan's new multiple range test and Student's two-tailed $t$-test. All $P$-values are based on two-sided hypothesis testing.

### RESULTS

### Effect of GH-RH antagonists on colon cancers in vivo

At the end of Experiments I and II, the average tumour volume in the groups receiving MZ-4-71 was reduced by about 45% ($P < 0.05$) compared to the control groups (Figure 1 A,B). Tumour weights were also decreased in the same groups in both experiments, however, the differences from control were not significant statistically (Table 1). In Experiment III, daily treatment with 20 µg day$^{-1}$ of MZ-5-156 or JV-1-36 strongly inhibited growth of tumours, reducing tumour volume by 57% and 58% and tumour weights by 57% and 43% respectively. The effect of JV-1-10 was weaker, and treatment with MZ-4-71 resulted only in a non-significant inhibition (Figure 1C). Tumour volume and weight data are shown in Table 1.

Histological studies demonstrated that treatment with MZ-4-71 significantly reduced the number of mitotic cells and AgNOR counts in tumours in Experiment I and II (Table 2). In Experiment II, the number of cells incorporating BrdU was reduced by 50% in the group treated with MZ-4-71. AgNOR numbers and BrdU indices of individual tumours showed a good correlation ($r$ = 0.75). In Experiment III, mitotic indices were lower in groups treated with GH-RH antagonist, and AgNORs were reduced significantly by all therapies. Apoptotic cells were identified by their characteristic morphology in histological slides stained with HE. Parallel electron microscopic investigations were helpful in recognizing apoptotic cells (Figure 2). The number of apoptotic cells was increased by MZ-4-71 in Experiment I and II, and by MZ-5-156 in Experiment III. The ratio of apoptotic to mitotic indices was significantly higher in the groups receiving MZ-4-71, MZ-5-156 and JV-1-36. The quantitative histological data are shown in Table 2.

### Receptor studies

In the control group of Experiment II, radiolabelled IGF-I was bound to a single class of high affinity ($K_d$ = 0.92 ± 0.04 nM), low capacity ($B_{max}$ = 129.7 ± 16.6 fmol mg$^{-1}$ membrane protein) binding sites (Figure 3). Treatment with GH-RH antagonist MZ-4-71 did not affect significantly the affinity ($K_d$ = 0.83 ± 0.10 nM), or capacity ($B_{max}$ = 135.9 ± 32.4 fmol mg$^{-1}$ protein) of the receptors for IGF-I in HT-29 tumour membrane fractions.

### IGF-I and IGF-II concentrations in tumour tissue

IGF-I concentration in the control tumours of Experiment II was 219 ± 4.2 pg 100 µg$^{-1}$ protein and 139 ± 12.7 pg 100 µg$^{-1}$ protein in the cancers treated with MZ-4-71. The difference was not significant statistically. IGF-II levels in HT-29 cancers were analysed in Experiments II and III. The results are shown in Table 1. IGF-II concentrations were decreased in the tumours treated with MZ-4-71 in Experiment II, and after therapy with MZ-5-156 and JV-1-36 in Experiment III.

### Expression of IGF-I and IGF-II mRNA in HT-29 cells and cancers

IGF-II mRNA in HT-29 cancers was expressed as IGF-II β-actin$^{-1}$ ratios. The results are shown in Table 1. Treatment with MZ-5-156 and JV-1-36 in Experiment III reduced IGF-II mRNA in colon

© 2000 Cancer Research Campaign

**Table 2** Effect of treatment with various GH-RH antagonists on some histological characteristics of HT-29 human colon cancers growing in nude mice

| Groups | Mitotic index | Apoptotic index | Ratio of apoptotic to mitotic indices | Number of AgNORs cell$^{-1}$ | BrdU index |
|---|---|---|---|---|---|
| Experiment I dose (see Table 1) | | | | | |
| 1. Control | 11.2 ± 1.4 | 7.3 ± 1.7 | 0.72 ± 0.21 | 7.20 ± 0.23 | |
| 2. MZ-4-71 | 6.9 ± 1.1[a] | 10.0 ± 1.1 | 1.56 ± 0.27[a] | 6.20 ± 0.39[a] | |
| 3. MZ-5-186 | 8.0 ± 1.7 | 6.1 ± 1.1 | 0.86 ± 0.20 | 6.40 ± 0.29 | |
| Experiment II dose (see Table 1) | | | | | |
| 1. Control | 16.5 ± 1.2 | 5.4 ± 0.6 | 0.33 ± 0.04 | 6.85 ± 0.16 | 140.0 ± 28.4 |
| 2. MZ-4-71 | 12.0 ± 1.3[b] | 10.0 ± 0.7[b] | 1.02 ± 0.17[b] | 5.60 ± 0.11[b] | 69.8 ± 7.4[a] |
| Experiment III dose (see Table 1) | | | | | |
| 1. Control | 14.1 ± 1.2 | 7.3 ± 1.3 | 0.57 ± 0.14 | 6.99 ± 0.15 | |
| 2. MZ-4-71 | 12.7 ± 1.2 | 8.9 ± 0.9 | 0.73 ± 0.09 | 6.16 ± 0.10[b] | |
| 3. MZ-5-156 | 9.1 ± 1.0[a] | 11.4 ± 1.3[a] | 1.40 ± 0.23[a] | 5.63 ± 0.10[b] | |
| 4. JV-1-10 | 10.6 ± 1.8 | 8.2 ± 1.0 | 0.87 ± 0.12 | 6.25 ± 0.13[b] | |
| 5. JV-1-36 | 7.4 ± 1.5[b] | 9.4 ± 1.1 | 1.33 ± 0.17[a] | 5.77 ± 0.15[b] | |

Values are means ± s.e.m., [a]$P < 0.05$, [b]$P < 0.01$.

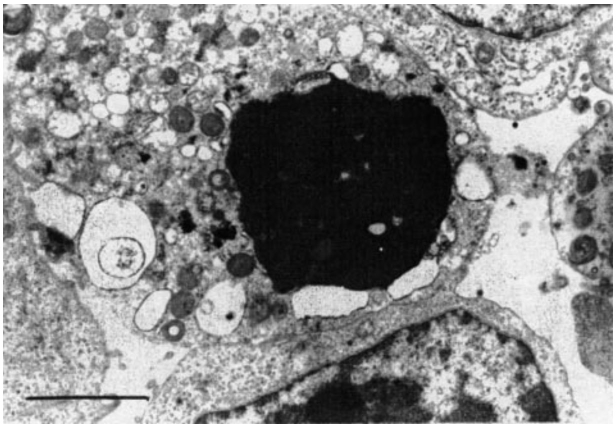

**Figure 2** Electron microscopic picture of an apoptotic cell showing dense shrunken nucleus and condensed cytoplasmic organelles in a HT-29 tumour after treatment with MZ-4-71 in Experiment II (Bar = 5 µm)

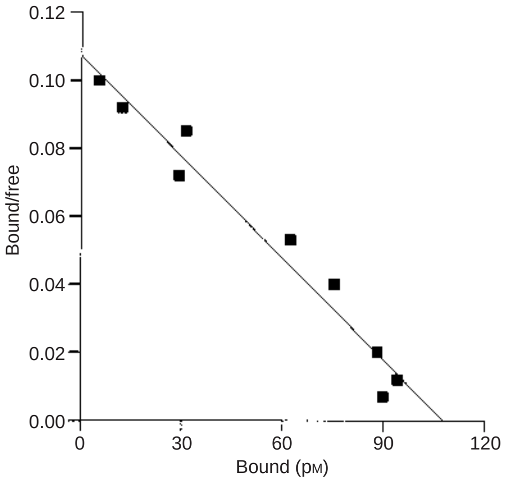

**Figure 3** Representative example of Scatchard plots of [$^{125}$I]IGF-I binding to the membrane fraction isolated from HT-29 human colon cancers. Specific binding was determined as described. Each point represents mean of triplicate determinations cancers ($P < 0.05$). A representative gel is shown in Figure 4. IGF-I mRNA could not be detected in HT-29 cancers.

### Serum GH, IGF-I and IGF-II levels

Levels of GH, IGF-I and IGF-II in serum were determined in Experiment II and III. In Experiment III, treatment with MZ-5-156 decreased serum IGF-I by 26%, other analogues did not affect serum IGF-I levels (data not shown). None of the analogues produced significant changes of serum IGF-II and GH.

### In vitro studies

Exogenous IGF-I at 10 and 25 ng ml$^{-1}$ and IGF-II at 500 and 800 ng ml$^{-1}$ concentrations enhanced growth of HT-29 cells cultured in serum containing medium, and the stimulatory effects of IGFs were more evident at 66 h (Figure 5). Addition of MZ-5-156 to the medium at $3 \times 10^{-6}$ M concentration inhibited proliferation of the colon cancer cells (Figure 5), the effects being significant after 114 h.

HT-29 cells were also grown in serum-free medium. The addition of 25 ng ml$^{-1}$ of IGF-I or 800 ng ml$^{-1}$ of IGF-II significantly increased cell growth after 66 h ($P < 0.05$). Slight stimulatory effects ($P < 0.05$) were also seen after 114 h. MZ-5-156 at $3 \times 10^{-6}$ concentration inhibited proliferation of the colon cancer cells at both time points (Figure 6).

IGF-I concentration in serum-free medium was not increased after seeding of HT-29 cells, and was not influenced by addition of MZ-5-156 or GH. However, IGF-II levels in serum-free medium showed dramatic increases in relation to the time of incubation after seeding of HT-29 cells, demonstrating that the cells secrete IGF-II into the medium. Adding of MZ-5-156 to the medium, dose-dependently reduced IGF-II levels at 138 h culture time to about 60% of control values (Figure 7).

### DISCUSSION

IGF-I is the mediator of the normal growth promoting effect of GH, and operates by activating the IGF-I receptor (IGF-I R) (Westley and May, 1995). The IGF-I R is required for cancer cell proliferation by establishing and maintaining the transformed

 *© 2000 Cancer Research Campaign*

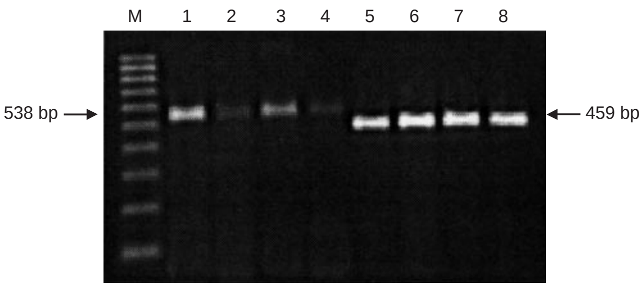

**Figure 4** RT-PCR analysis of human IGF-II and β-actin mRNA expression in HT-29 colon cancers. The sizes of expected PCR products were 538 and 459 for hIGF-II and hβ-actin respectively. Lanes: M: molecular weight marker (Sigma); 1–4: IGF-II; 5–8: β-actin. Lanes 1 and 5: control, 2 and 6: MZ-5-156, 3 and 7: MZ-4-71, 4 and 8: JV-1-36

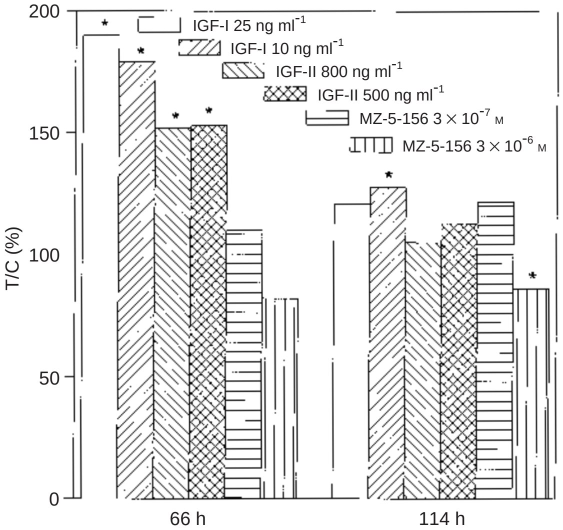

**Figure 5** Effect of IGF-I, IGF-II and GH-RH antagonist MZ-5-156 on growth of HT-29 human colon cancer cells cultured in serum-containing medium 66 and 114 h after addition of the peptides. Growth was measured by crystal violet assay and expressed as percentage of control. **P* < 0.05 vs control

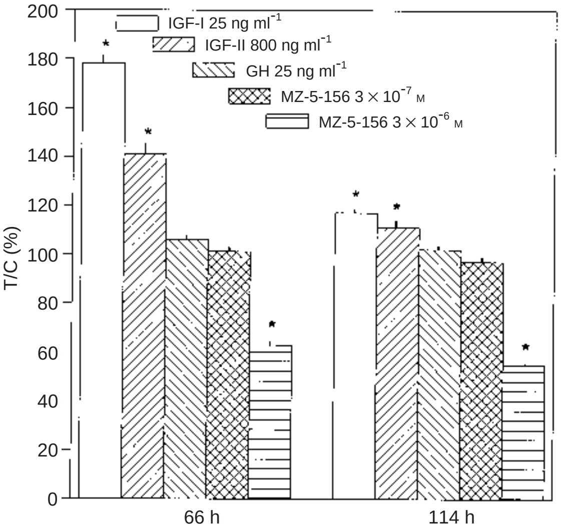

**Figure 6** Effect of IGF-I, IGF-II, GH and GH-RH antagonist MZ-5-156 on growth of HT-29 human colon cancer cells cultured in serum-free medium 66 and 114 h after addition of the peptides. Growth was measured by crystal violet assay and expressed as percentage of control. **P* < 0.05 vs control

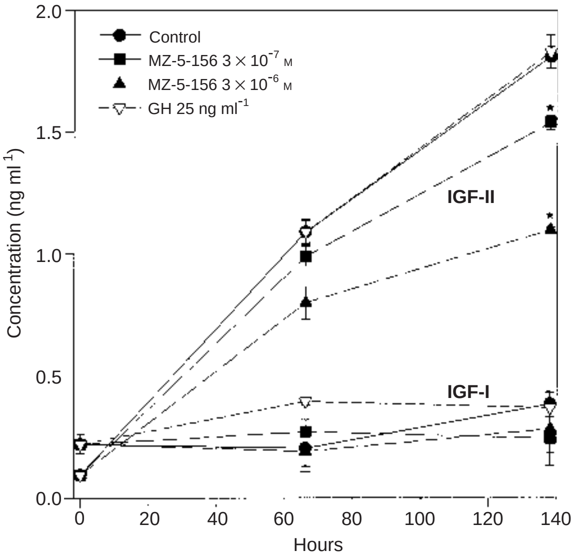

**Figure 7** Concentration of IGF-I and IGF-II in serum-free medium 66 and 138 h after addition of GH-RH antagonist MZ-5-156 or GH (at 0 h). HT-29 cells were seeded 24 h before addition of peptides. Control cultures received serum-free medium only. All IGF-II levels were higher at 66 h and 138 h than those at 0 h (*P* < 0.001). **P* < 0.01 vs control at 138 h phenotype (Baserga, 1995). Overexpression of IGF-IRs is sufficient for cell transformation and increased cell proliferation (Blakesley et al, 1997). Genetically engineered dominant negative human IGF-IR induces apoptosis and inhibits cell growth and tumorigenesis (D'Ambrosio et al, 1996).

The IGF-IR is also target for IGF-II. IGF-II has an important role in progression of various tumours (Singh and Rubin, 1993; Toretsky and Helman, 1996; Schally et al, 1998). Stimulation of IGF-IR by IGF-II through autocrine or paracrine pathways (Toretsky and Helman, 1996) may allow cancer cells to maintain unregulated growth and resist programmed cell death (Singleton et al, 1996).

More and more findings are accumulating about the role of IGF-II in progression of colorectal cancers (Tricoli et al, 1986; Lahm et al, 1992, 1996; Toretsky and Helman, 1996). IGF-II levels are higher in blood of patients with colorectal cancer compared with matching normal population, but IGF-I concentrations were found normal (elAtiq et al, 1994). IGF-II was found to be a growth factor for some colorectal cancer cell lines (Lahm et al, 1992). IGF-I receptor was expressed in all 12 colon cancer cell lines tested, and antibody to the IGF-IR inhibited growth of these lines (Lahm et al, 1996). Williams et al (1992) also demonstrated the significance of IGF-I in development of colon cancers, and the growth of several colon cancer cell lines in serum-free medium was stimulated by IGF-I.

In view of the essential role of IGFs in tumour progression, the regulatory mechanisms need to be clarified. According to the classical hypotheses, IGF-I may function as a circulating hormone secreted by the liver in response to GH (Jones and Clemmons, 1995). GH may also regulate local production of IGF-I in other

© 2000 Cancer Research Campaign 

tissues. However, the regulation of IGF-II is less clear and involvement of GH in IGF-II production is controversial (Jones and Clemmons, 1995; Schally et al, 1998). The effects of IGFs are also regulated by IGF-binding proteins (IGFBP). IGFs bind to these proteins with higher affinity than to the IGF-I receptor and thus IGFBPs can modulate their bioavailability for tissues (Mohan and Baylink, 1996; Toretsky and Helman, 1996).

In the present study, various GH-RH antagonists inhibited growth of HT-29 colon cancer xenografts with variable efficacy. MZ-5-186 and JV-1-10 had only a small effect. MZ-4-71 was more powerful when it was given twice daily, while MZ-5-156 and JV-1-36 caused the strongest inhibition. These differences in activity can be explained by different stability of the compounds in circulation (Zarandi et al, 1994, 1997; Kovacs et al, 1996, 1997). It was demonstrated previously that MZ-5-156 has a longer life-span in blood than MZ-4-71 (Kovacs et al, 1996, 1997), thus given once a day, MZ-4-71 is less effective than when administered twice daily.

Histological analysis demonstrated that decreased cell proliferation and increased apoptosis both contributed to tumour inhibition by the GH-RH antagonists. IGFs and IGF-IR have special anti-apoptotic activity. In our studies, the decrease in IGF-II activity caused by the GH-RH antagonists resulted in a restoration of apoptotic process that contributed to inhibition of tumour growth.

The mechanism of action of the GH-RH antagonists is not fully clarified, but our present in vivo and in vitro studies shed additional light on the tumour inhibitory effects of these peptides. There were no changes in IGF-I levels in blood showing that tumour inhibition can occur without a decrease in the systemic supply of hepatic IGF-I. GH-RH antagonists did not affect binding characteristics of IGFRs or IGF-I concentration in the tumours. In contrast, IGF-II levels were significantly decreased in the HT-29 cancers of mice treated with MZ-4-71, MZ-5-156 or JV-1-36, and mRNA for IGF-II was also reduced in the same tumours.

In vitro studies showed that HT-29 cells produce and secrete IGF-II but not IGF-I. These findings are in accord with the results of other studies (Lobie et al, 1990; Singh and Rubin, 1993; elAtiq et al, 1994). Exogenous IGF-I and IGF-II enhanced growth of HT-29 cells in our experiments. Lahm et al (1992, 1996) reported similar results, whereas others could not detect any effects of exogenous IGFs on colon cancer cell growth (Singh and Rubin, 1993). In view of the key role of IGF-II in cancers and the fact that IGF-II acts through the IGF-I receptor, either of these may become targets for new therapeutic approaches to colorectal cancer.

GH-RH antagonists might act by inhibiting GH/IGF-I axis, or may have a direct effect on tumours. The peptide receptors in tumours that respond to GH-RH antagonists appear to be different from the pituitary GH-RH receptors (unpublished data). GH-RH is a member of the family of peptides that includes vasoactive intestinal peptide (VIP), glucagon, secretin, gastric inhibitory peptide, and pituitary adenylate cyclase-activating peptide (PACAP) (reviewed in Lamharzi et al, 1998). These peptides demonstrate significant amino acid sequence homology. The human and rat GH-RH receptors are homologous to secretin and VIP receptor proteins (Mayo, 1992). Thus, GH-RH and its analogues may act through other related receptors. VIP receptors and PACAP receptors were detected in various gastrointestinal tumours (Reubi, 1995; Raderer et al, 1998) and also in human colon cancer cell lines including HT-29 cancers (Lelievre et al, 1998). Antagonistic analogues of GH-RH suppress the stimulatory effects not only of GH-RH but also of VIP on the cAMP production of various cancer cells (Csernus et al, 1999). In our in vitro studies, GH-RH antagonist MZ-5-156 inhibited the proliferation of HT-29 cells growing in normal or serum-free medium. MZ-5-156 also decreased the secretion of IGF-II by HT-29 cells into the medium, thus suggesting a receptor-based mechanism for tumour growth inhibition. Since the GH-RH antagonist exerted these effects in vitro, this reinforces the hypothesis of direct action of MZ-5-156 on HT-29 cells.

Investigations are in progress to identify the peptide receptors that bind GH-RH antagonists in various tumours. It is probable that these receptors belong to the common neuropeptide receptor family (Mayo, 1992) that can bind peptides with similar structures such as VIP or GH-RH. After a future identification of binding sites for GH-RH analogues some intracellular pathways of their action may be clarified. The potent anti-tumour activity of GH-RH antagonists supports the merit of further investigation of their mechanisms of action and the development of more active and stable analogues.

Our work indicates that GH-RH antagonists by virtue of inhibiting IGF-I and/or II may offer new approaches to treatment of colorectal cancer and other malignancies.

### ACKNOWLEDGEMENTS

We thank Esther Li, Veronica Lea, Elena Glotser and Ilona Hoffmann for their technical assistance. The gift of materials for RIA provided by Dr AF Parlow, Dr LE Underwood and Dr J van Wyk from the National Institute of Diabetes and Digestive and Kidney Diseases (NIDDKs) National Hormone and Pituitary Program are greatly appreciated. This work was partially supported by a grant from ASTA Medica (Frankfurt on Main, Germany) to Tulane University School of Medicine and by the Medical Research Service of the Veterans Affairs Departments (all of AVS).

### REFERENCES

Baserga R (1995) The insulin-like growth factor I receptor: a key to tumour growth. *Cancer Res* **55**: 249–252

Bernhardt G, Reile H, Birnbock H, Spruss T and Shonenberger H (1992) Standardized kinetic microassay to quantify differential chemosensitivity on the basis of proliferative activity. *J Cancer Res Clin Oncol* **118**: 35–43

Blakesley VA, Stannard BS, Kalebic T, Helman LJ and LeRoith D (1997) Role of the IGF-I receptor in mutagenesis and tumour promotion. *J Endocrinol* **152**: 339–344

Csernus V, Schally AV and Groot K (1999) Antagonistic analogs of growth hormone-releasing hormone (GHRH) inhibit cyclic AMP production of human cancer cell lines in vitro. Peptides **20**: 843–850

D'Ambrosio C, Ferber A, Resnicoff M and Baserga R (1996) A soluble insulin-like growth factor I receptor that induces apoptosis of tumour cells in vivo and inhibits tumorigenesis. *Cancer Res* **56**: 4013–4020

elAtiq F, Garrouste F, Remacle-Bonnet M, Sastre B and Pommier G (1994) Alterations in serum levels of insulin-like growth factors and insulin-like growth-factor-binding proteins in patients with colorectal cancer. *Int J Cancer* **57**: 491–497

Garrouste FL, Remacle-Bonnet MM, Lehmann NM-A, Marvaldi JL and Pommier GJ (1997) Up-regulation of insulin/insulin-like growth factor-I hybrid receptors during differentiation of HT29-D4 human colonic carcinoma cells. *Endocrinology* **138**: 2021–2032

Hardcastle JD (1997) Colorectal cancer. CA-A Cancer J Clin 47: 66–69

Jones JI and Clemmons DR (1995) Insulin-like growth factors and their binding proteins: biological actions. *Endocrine Rev* **16**: 3–34

Kiaris H and Schally AV (1999) Decrease in telomerase activity in U-87MG human glioblastomas after treatment with an antagonist of growth hormone-releasing hormone. *Proc Natl Acad Sci USA* **96**: 226–231

Kovacs M, Zarandi M, Halmos G, Groot K and Schally AV (1996) Effects of acute and chronic administration of a new potent antagonist of growth hormone-releasing hormone in rats: Mechanisms of action. *Endocrinol* **137**: 5364–5369

 *© 2000 Cancer Research Campaign*

Kovacs M, Schally AV, Zarandi M and Groot K (1997) Inhibition of GH release in rats by new potent antagonists of growth hormone-releasing hormone (GH-RH). *Peptides* **18**: 431–438

Labianca R, Pessi MA and Zamparelli G (1997) Treatment of colorectal cancer. *Drugs* **53**: 593–607

Lahm H, Suardet L, Laurent PL, Ficher JR, Ceyhan A, Givel J-C and Odartchenko N (1992) Growth regulation and co-stimulation of human colorectal cancer cell lines by insulin-like growth factor I, II and transforming growth factor α. *Br J Cancer* **65**: 341–346

Lahm H, Amstad P, Yilmaz A, Fischer JR, Givel JC, Odartchenko N and Sordat B (1996) Differential effect of interleukin-4 and transforming growth factor beta 1 on expression of proto-oncogenes and autocrine insulin-like growth factor II in colorectal carcinoma cells. *Biochem Biophys Res Commun* **220**: 334–340

Lamharzi N, Schally AV, Koppan M and Groot K (1998) Growth hormone-releasing hormone antagonist MZ-5-156 inhibits growth of DU-145 human androgen-independent prostate carcinoma in nude mice and suppresses the levels and mRNA expression of insulin-like growth factor II. *Proc Natl Acad Sci USA* **95**: 8864–8868

Lelievre V, Meunier AC, Caigneaux E, Falcon J and Muller JM (1998) Differential expression and function of PACAP and VIP receptors in four human colonic adenocarcinoma cell lines. *Cell Signal* **10**: 13–26

Lobie PE, Breipohl W and Waters MJ (1990) Growth hormone receptor expression in the rat gastrointestinal tract. *Endocrinology* **126**: 299–306

Ma J, Pollak MN, Giovannucci E, Chan JM, Tao Y, Hennekens CH and Stampfer MJ (1999) Prospective study of colorectal cancer risk in men and plasma levels of insulin-like growth factor (IGF)-I and IGF-binding protein-3. *J Natl Cancer Inst* **91**: 620–625

Macaulay VM (1992) Insulin-like growth factors and cancer. *Br J Cancer* **65**: 311–320

Magnusson BA, Raju RVS, Moyana TN and Sharma RK (1995) Increased N-myristoyl-transferase activity observed in rat and human colonic tumours. *J Natl Cancer Inst* **87**: 1630–1635

Mayo KE (1992) Molecular cloning and expression of a pituitary-specific receptor for growth hormone-releasing hormone. *Molec Endocrin* **6**: 1734–1744

Michell NP, Langman MJS and Eggo MC (1997) Insulin-like growth factors and their binding proteins in human colonocytes: preferential degradation of insulin-like growth factor binding protein 2 in colonic cancers. *Brit J Cancer* **76**: 60–66

Mohan S and Baylink DJ (1996) Editorial: Insulin-like growth factor (IGF)-binding proteins in serum – do they have additional roles besides modulating the endocrine IGF actions? *J Clin Endocrinol Metab* **81**: 3817–3820

Munson PJ and Rodbard D (1980) Ligand: a versatile computerized approach for characterization of ligand-binding systems. *Anal Biochem* **107**: 220–239

Orme SM, McNally RJQ, Cartwright RA and Belchetz PE (1998) Mortality and cancer incidence in acromegaly: A retrospective cohort study. *J Clin Endocrinol Metab* **83**: 2730–2734

Pinski J, Schally AV, Groot K, Halmos G, Szepeshazi K, Zarandi M and Armatis P (1995) Inhibition of growth of human osteosarcomas by antagonists of growth hormone-releasing hormone. *J Natl Cancer Inst* **87**: 1787–1794

Pollak MN and Schally AV (1998) Mechanism of antineoplastic action of somatostatin analogs. *Proc Soc Exp Biol Med* **217**: 143–152

Pollak MN, Perdue JF, Margolese RG, Baer K and Richard M (1987) Presence of somatomedin receptors on primary human breast and colon carcinomas. *Cancer Lett* **38**: 223–230

Pollak M, Polychronakos C and Guyda H (1989) Somatostatin analogue SMS 201–995 reduces serum IGF-I levels in patients with neoplasms potentially IGF-I dependent. *Anticancer Research* **9**: 889–891

Raderer M, Kurtaran A, Hejna M, Vorbeck F, Angelberger P, Scheithauer W and Virgolini I (1998) $^{123}$I-labelled vasoactive intestinal peptide receptor scintigraphy in patients with colorectal cancer. *Br J Cancer* **78**: 1–5

Reubi JC (1995) In vitro identification of vasoactive intestinal peptide receptors in human tumours: implications for tumour imaging. *J Nucl Med* **36**: 1846–1853

Schally AV (1994) Hypothalamic hormones: from neuroendocrinology to cancer therapy. *Anti-Cancer Drugs* **5**: 115–130

Schally AV, Kovacs M, Toth K and Comaru-Schally AM (1998) Antagonistic analogs of growth hormone-releasing hormone (GH-RH): endocrine and oncological studies. In: Bercu BB, Walker RF, eds. Growth hormone secretagogues in clinical practice. New York: Marcel Dekker; 145–162

Singh P and Rubin N (1993) Insulinlike growth factors and binding proteins in colon cancer. *Gastroenterology* **105**: 1218–1237

Singleton JR, Randolph AE and Feldman EL (1996) Insulin-like growth factor I receptor prevents apoptosis and enhances neuroblastoma tumorigenesis. *Cancer Res* **56**: 4522–4529

Szepeshazi K, Korkut E and Schally AV (1991) Decrease in the AgNOR number in Dunning R3327 prostate cancers after treatment with an agonist and antagonist of luteinizing hormone-releasing hormone. *Am J Pathol* **138**: 1273–1277

Toretsky JA and Helman LJ (1996) Involvement of IGF-II in human cancer. *J Endocrinol* **149**: 367–372

Tricoli JV, Rall LB, Karakousis CP, Herera L, Petrelli NJ, Bell GI and Shows TB (1986) Enhanced levels of insulin-like growth factor messenger RNA in human colon carcinomas and liposarcomas. *Cancer Res* **46**: 6169–6173

Varga JL, Schally AV, Csernus VJ, Zarandi M, Halmos G, Groot K and Rekasi Z (1999) Synthesis and biological evaluation of antagonists of growth hormone-releasing hormone with high and protracted in vivo activities. *Proc Natl Acad Sci (USA)* **96**: 692–697

Westley BR and May FEB (1995) Insulin-like growth factors: the unrecognised oncogenes. *Br J Cancer* **72**: 1065–1066

Williams NN, Gyorfi T, Iliopoulos D, Herlyn D, Greenstein D, Linnenbach AJ, Daly JM, Jensen P, Rodeck U and Herlyn M (1992) Growth-factor-independence and invasive properties of colorectal carcinoma cells. *Int J Cancer* **50**: 274–280

Zarandi M, Horvath JE, Halmos G, Pinski J, Nagy A, Groot K, Rekasi Z, Schally AV (1994) Synthesis and biological activities of highly potent antagonists of growth hormone-releasing hormone. *Proc Natl Acad Sci USA* **91**: 12298–12301

Zarandi M, Kovacs M, Horvath JE, Toth K, Halmos G, Groot K, Nagy A, Kele Z, Schally AV (1997) Synthesis and in vitro evaluation of new potent antagonists of growth hormone-releasing hormone (GH-RH). *Peptides* **18**: 423–430

© 2000 Cancer Research Campaign 
